# Radiotherapy for glioblastoma patients with poor performance status

**DOI:** 10.1007/s00432-021-03770-9

**Published:** 2021-08-26

**Authors:** Christina Schröder, Dorothee Gramatzki, Erwin Vu, Matthias Guckenberger, Nicolaus Andratschke, Michael Weller, Caroline Hertler

**Affiliations:** 1 Department of Radiation Oncology and Competence Center for Palliative Care, University Hospital Zurich, University of Zurich, Rämistrasse 100, 8091 Zurich, Switzerland; 2 Department of Neurology, University Hospital Zurich, University of Zurich, Zurich, Switzerland

**Keywords:** Glioblastoma, Radiation, Performance status, Survival

## Abstract

**Purpose:**

There is limited information on treatment recommendations for glioblastoma patients with poor performance status. Here, we aim to evaluate the association of radiotherapy on survival in glioblastoma patients presenting with poor postoperative performance status in first-line setting.

**Methods:**

We retrospectively analyzed data of 93 glioblastoma patients presenting with poor postoperative performance status (ECOG 2–4) at the University Hospital Zurich, Switzerland, in the years 2005–2019. A total of 43 patients received radiotherapy with or without systemic therapy in the first-line setting, whereas 50 patients received no additive local or systemic treatment after initial biopsy or resection. Overall survival was calculated from primary diagnosis and from the end of radiotherapy. In addition, factors influencing survival were analyzed.

**Results:**

Median overall survival from primary diagnosis was 6.2 months in the radiotherapy group (95% CI 6.2–14.8 weeks, range 2–149 weeks) and 2.3 months in the group without additive treatment (95% CI 1.3–7.4 weeks, range 0–28 weeks) (*p* < 0.001). This survival benefit was confirmed by landmark analyses. Factors associated with overall survival were extent of resection and administration of radiotherapy with or without systemic treatment. Median survival from end of radiotherapy was 3 months (95% CI 4.3–21.7 weeks, range 0–72 weeks), with 25.6% (*n* = 11) early termination of treatment and 83.7% (*n* = 36) requiring radiotherapy as in-patients. Performance status improved in 27.9% (*n* = 12) of patients after radiotherapy.

**Conclusion:**

In this retrospective single-institution analysis, radiotherapy improved overall survival in patients with poor performance status, especially in patients who were amendable to neurosurgical resection.

**Supplementary Information:**

The online version contains supplementary material available at 10.1007/s00432-021-03770-9.

## Introduction

Eighteen to 37% of glioblastoma patients present in a poor pre- or postoperative performance status at the time of diagnosis (Chang et al. 2003; Lutterbach et al. 2003; Bauchet et al. 2010; Gulati et al. 2011; Scott et al. 2012; Stark et al. 2012; Badaoui et al. 2014; McNamara et al. 2014; Zouaoui et al. 2014; Gramatzki et al. 2017). Performance status, age, mental status and extent of surgical resection have been confirmed as important prognostic factors in these patients, and low performance status at diagnosis indicates a particularly poor prognosis (Curran et al. 1993; Li et al. 2011; Scott et al. 2012).

The standard treatment of glioblastoma consists of surgery followed by radiotherapy in 30 fractions and concurrent and maintenance systemic therapy with temozolomide (TMZ) (Stupp et al. 2005; Wen et al. 2020; Weller et al. 2021). However, patients in poor performance status or elderly patients (> 70 years), are usually treated with adapted treatment regimens such as short course radiotherapy with or without chemotherapy, or alkylating chemotherapy only in O^6^-methylguanine-DNA methyltransferase (*MGMT*) promotor methylated patients (Roa et al. 2004; Malmstrom et al. 2012; Wick et al. 2012; Perry et al. 2017). Likewise, almost all clinical trials with experimental drugs preclude the enrolment of patients with poor performance status.

For elderly patients in good performance status, a survival benefit from radiotherapy after surgical resection of different extent has been confirmed (Keime-Guibert et al. 2007). However, data regarding the treatment of patients in poor performance status is scarce, especially considering that those patients are usually excluded from clinical trials, which minimizes the availability of data on potential benefit or harm for this selected patient group. A trial of 70 patients with a median Karnofsky performance status (KPS) of 60% compared the benefit of temozolomide therapy only to a historical control of patients treated with best supportive care. Temozolomide alone was characterized by acceptable tolerability especially in patients with a methylated MGMT-promotor and was associated with an improvement of the functional status. However, radiotherapy was not part of this study (Gallego Perez-Larraya et al. 2011).

To further evaluate a potential benefit of radiotherapy in patients with low performance status at diagnosis, we retrospectively analyzed the data of 43 patients with poor performance status receiving additive radiotherapy, either alone or in combination with chemotherapy, and of a control group of 50 patients receiving a diagnostic biopsy, partial or gross total resection without further tumor-targeted therapy.

## Material and methods

### Data source

We conducted a retrospective single center cohort study of glioblastoma patients > 18 years at diagnosis, treated at the University Hospital Zurich between April 2005 and June 2019, with poor postoperative performance status (ECOG PS 2–4) after biopsy or neurosurgical resection in the first-line setting. Electronic patient files and pathology reports were the main source of data acquisition. The study was conducted in accordance with the Declaration of Helsinki and applicable regulatory requirements and has been approved by the local ethics committee (KEK-ZH-Nr. 2009-0135/1; KEK-ZH-Nr. 2015-0437).

### Disease and treatment characteristics

All tumors were classified according to the World Health Organization (WHO) 2007 criteria in the local pathology department, for patients diagnosed before 2016, and according to the WHO 2016 criteria for patients diagnosed after that time. In a second step all tumors classified by the 2007 criteria were re-classified by IDH mutation status in accordance with the WHO 2016 classification when sufficient tissue was available. IDH1/2 status was obtained by immunohistochemistry or sequencing analysis; *MGMT* promotor methylation status was determined by methylation-specific PCR. Extent of resection was assessed based on early postoperative imaging reports in all patients. For treatment planning, planning CT scans as well as MRI were used. The gross tumor volume (GTV) was contoured as the primary tumor (if biopsy only) or the resection cavity including contrast-enhanced regions. A safety margin of 1.5 cm was added to derive the clinical target volume (CTV). An additional planning target volume (PTV) margin was added to compensate for setup errors. For grading of toxicity, CTCAE V. 5.0 was used.

### Statistical analysis

Overall survival was calculated according to the Kaplan–Meier method. Overall survival from the time of surgery (biopsy or resection) to death or from end of radiotherapy to death were calculated. Additional factors were analyzed using the log-rank test. Univariate and multivariate analyses were done using Cox regression. The multivariate model was applied to all patients who had complete information on all tested co-variables. For statistical analysis of patient characteristics and performance status, the Chi-squared test and the Fisher’s exact test were used. For statistical analysis, SPSS Version 25 was used (SPSS IBM Corp., Armonk, NY, USA).

## Results

### Patient characteristics

We evaluated the data of 93 patients with glioblastoma and poor postoperative performance status (ECOG 2–4) that were treated at our institution between April 2005 and June 2019. Median age of patients was 69 years in the radiotherapy group and 73 in the control group (*p* = 0.236). Median postoperative ECOG was 2 for both the radiotherapy group and the control group. A total of 43 patients received additive radiotherapy, 18 of which after partial or gross total resection (GTR) and the remaining after tumor biopsy only. Most patients in the radiotherapy group were treated with 15 × 2.67 Gy (*n* = 35; 81.4%), 9.3% (*n* = 4) were treated with 10 × 3 Gy and 9.3% (*n* = 4) were treated with other fractionations. Additional concurrent or sequential systemic therapy was given in 19 patients, most frequently temozolomide during and/or after radiotherapy (*n* = 14; 32.6%). The patients in the control group received no tumor-specific treatment after surgical resection or biopsy (best supportive care). Further patient and treatment characteristics are summarized in Table 1 and in the supplementary file.

**Table 1:** Patient characteristics

	Radiotherapy group		Control group		*p* value	Total
	*N*	%	*N*	%		*N*
Age (years)						
Median	69		73		0.236	
Range	56–85		29–90			
Sex						
Female	19	44.2	22	44.0	0.986	41
Male	24	55.8	28	56.0		52
ECOG						
2	29	31.2	28	30.1	0.626	57
3	13	14.0	20	21.5		33
4	1	1.1	2	2.2		3
IDH						
Wildtype	38	88.4	45	90.0	0.129	83
Mutated	2	4.7	0	0.0		2
Unknown	3	7.0	5	10.0	–	8
MGMT status						
Methylated	7	16.3	9	18.0	0.257	16
Unmethylated	23	53.5	15	30.0		38
Unknown	13	30.2	26	52.0	–	39
Surgery^a^						
Biopsy only	25	58.1	26	52.0	0.011	51
Partial resection	7	16.3	20	40.0		27
Gross total resection	11	25.6	4	8.0		15
Tumor localisation _1						
Deep structures	9	20.9	13	26	0.197	22
Frontal lobe	12	27.9	12	24		24
Temporal lobe	11	25.6	7	14		18
Parietal lobe	8	18.6	5	10		13
Occipital lobe	3	7.0	10	20		13
Infratentorial	0	0	2	4		2
Multifocal	0	0	1	2		1
Tumor localisation _2						
Bihemispheric	3	7.0	10	20	0.117	13
Left hemisphere	17	39.5	13	26		30
Right hemisphere	23	53.5	25	50		48
Infratentorial	0	0	2	4		2
Planned radiation therapy						
15 × 2.67 Gy	35	81.4	n.a	n.a		35
10 × 3 Gy	4	9.3	n.a	n.a		4
Other	4	9.3	n.a	n.a		4
Systemic treatment (part of first-line treatment)						
None	24	55.8	n.a	n.a		24
TMZ before RT	3	7.0	n.a	n.a		3
TMZ during and/or after RT	14	32.6	n.a	n.a		14
CCNU after RT	2	4.7	n.a	n.a		2
Bevacizumab after RT	7	16.3	n.a	n.a		7

^*a*^Sign difference RT group and control group (*p* < 0.05, Chi-Squared test)

### Adherence and practical aspects of radiation treatment

Compliance to radiotherapy was high with 32 patients (74.4%) receiving the planned dose of radiotherapy. In 11 patients (25.6%) treatment was terminated early because of a decline in performance status, either as the result of disease progression or due to pre-existing morbidity. A total of 36 patients (83.7%) received radiation treatment as in-patients for at least a part of the treatment. In 14 patients (32.6%) a specialized palliative care team was involved at some point during radiotherapy treatment. Radiotherapy was well tolerated with no ≥ G3 toxicities.

### Overall survival

**Fig. 1:**
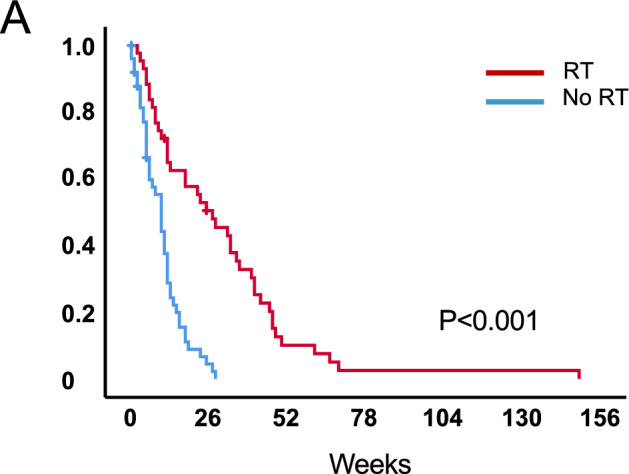
Overall survival in patients with newly diagnosed glioblastoma and ECOG $\ge$ 2 stratified by treatment vs. no treatment **a**

**Fig. 2:**
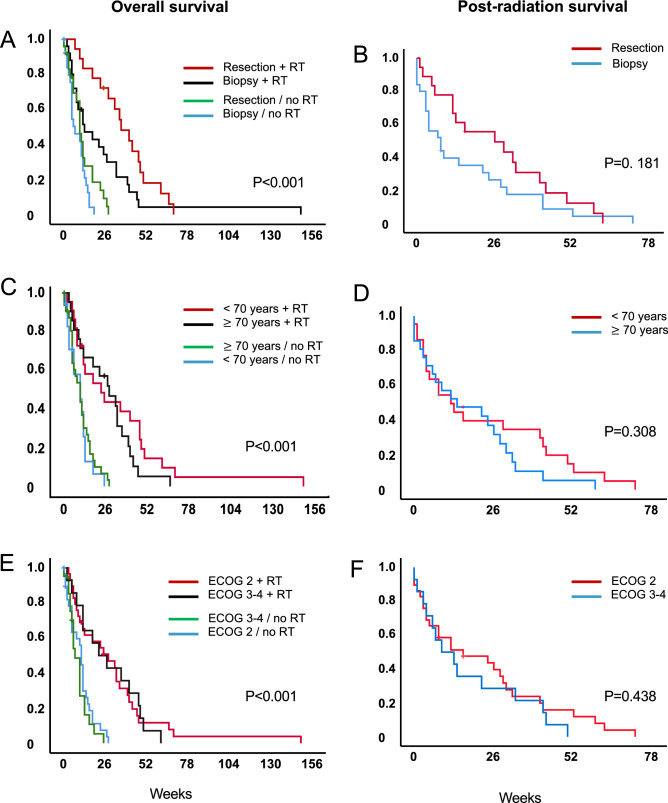
Overall survival of the whole population (left) and survival from end of RT in the treatment group (right) were stratified by extent of resection (resection vs. biopsy) **a**, **b**; age group (< 70 years vs $\ge$ 70 years) **c**, **d**; and ECOG at diagnosis (2 vs. 2–3) **e**, **f**

**Fig. 3:**
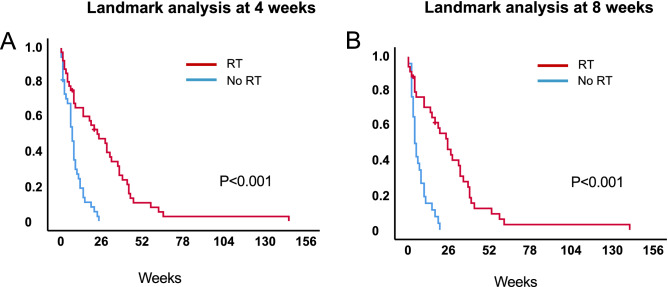
Overall survival of the whole population stratified by treatment vs. no treatment. Landmark analyses were performed at 4 weeks **a** and 8 weeks **b**

At the time of analysis, median follow-up of living patients was 2 weeks (95% CI 0.0–6.8) and six patients were still alive. The median survival after primary diagnosis was 27 weeks (95% CI 6.2–14.8, range 2–149) for patients treated with radiotherapy and 10 weeks (95% CI 1.3–7.4, range 0–28) for the patients in the control group (Fig. 1A) (*p* < 0.001). Patients that received a biopsy only had a median survival from diagnosis of 13 weeks (95% CI 2.6–23.4, range 2–149) when treated with radiotherapy (*n* = 25) and 7 weeks (95% CI 3.4–10.6, range 0–19) with best supportive care (*n* = 26) (Fig. 2A) (*p* < 0.001). Older patients ≥ 70 years had a median overall survival of 28 weeks (95% CI 14.1–41.9, range 3–66) when treated with radiotherapy, and 10 weeks in the best supportive care group (95% CI 6.0–14.0, range 0–28) (Fig. 2C) (*p* < 0.001). When separated by ECOG, patients with poor ECOG (3–4) showed a median overall survival of 22 weeks (95% CI 5.5–38.5, range 2–61) when treated with radiotherapy versus 7 weeks with best supportive care (95% CI 4.4–9.6, range 0–25 weeks) (Fig. 2E) (*p* < 0.001) (Table 2). In addition, a 4- and 8-week landmark analysis was performed to estimate OS without immortal time bias (Fig. 3A, B) showing similar results compared to analysis at diagnosis. Extent of surgical resection (*p* = 0.037) and postoperative treatment [either radiotherapy only (*p* = 0.002) or in combination with systemic therapy (*p* < 0.001)] showed statistical significance in univariate analysis and remained significant in multivariate analysis (Tables 3, 4).

**Table 2:** Survival data (log rank analysis)

	From diagnosis				From RT			
Survival	*N*	Median OS (weeks)	95% CI	*p* value	*N*	Median OS (weeks)	95% CI	*p* value
All patients	93	12	9.9–14.1		43	13	4.3–21.7	
RT								
Yes	43	27	6.2–14.8	< 0.001		n.a	n.a	n.a
No	50	10	1.3–7.4					
Age								
< 70 with RT	22	23	5.4–40.6	< 0.001	22	12	3.1–20.9	0.308
< 70 no RT	18	10	3.7–16.3					
≤ 70 with RT	21	28	14.1–41.9		21	14	0.0–32.7	
≥ 70 no RT	32	10	6.0–14.0					
ECOG								
2 with RT	29	28	10.8–45.2		29	16	0–35.7	0.438
2 no RT	29	11	9.3–12.7					
3–4 with RT	14	22	5.5–8.5		14	9	0–26.7	
3–4 no RT	21	7	4.4–9.6					
Surgery								
Biopsy with RT	25	13	2.6–23.4	< 0.001	25	8	1.5–14.5	0.181
Biopsy no RT	26	7	3.4–10.6					
PR/GTR with RT	18	36	24.2–47.8		18	26	1.4–50.6	
PR/GTR no RT	24	10	7.3–12.7

**Table 3:** Univariate analysis with regards to death (Cox regression analysis)

	*N* (events)	HR (95% CI)	*p* value
Age			
< 70 years	40 (37)	0.74 (0.48–1.16)	0.190
≥ 70 years	53 (50)	ref	
Gender			
Male	52 (48)	ref	
Female	41 (39)	1.13 (0.74–1.74)	0.565
ECOG			
2	58 (54)	0.87 (0.56–1.35)	0.527
3/4	35 (33)	ref	
Extent of resection			
Biopsy	51 (48)	1.58 (1.03–2.43)	0.037
Resection (partial/gross total resection)	42 (39)	ref	
MGMT promoter methylation status			
Unmethylated	38 (37)	ref	
Methylated	16 (13)	0.91 (0.49–1.70)	0.766
Treatment			
No tumor-specific treatment	50 (46)	ref	
Radiotherapy	24 (24)	0.42 (0.24–0.74)	0.002
Radiotherapy plus systemic treatment	19 (17)	0.13 (0.06–0.26)	< 0.001

**Table 4:** Multivariate analysis with regards to death (Cox regression analysis)

	*N* (events)	HR (95% CI)	*p* value
All patients	54 (51)		
Age			
< 70 years	26	1.40 (0.69–2.81)	0.352
≥ 70 years	28	Ref	
Gender			
Male	25	Ref	
Female	29	2.87 (1.46–5.67)	0.002
ECOG			
2	33	0.94 (0.52–1.70)	0.839
3/4	21	Ref	
Extent of resection			
Biopsy	25	3.93 (1.94–7.93)	< 0.001
Resection (partial/gross total resection)	29	Ref	
MGMT promoter methylation status			
Unmethylated	38	Ref	
Methylated	16	0.84 (0.43–1.62)	0.600
Treatment			
No tumor-specific treatment	24	Ref	
Radiotherapy	14	0.26 (0.12–0.59)	0.001
Radiotherapy plus systemic treatment	16	0.09 (0.03–0.25)	< 0.001

### Post-radiation survival

The median survival after radiotherapy (post-radiation survival) was 13 weeks (3 months, 95% CI 4.3–21.7 weeks, range 0–72 weeks). Twelve patients (27.9%) treated with radiotherapy died within 4 weeks after radiotherapy, 10 of which had a biopsy only (83.3%). Post-radiation survival did not differ significantly when stratified for extent of resection, age group or ECOG at diagnosis (Fig. 2B, D, F) (Table 2).

### Performance status

After RT, the performance status was improved at any follow-up appointment during the first 3 months in 12 patients (27.9%). The median increase in performance status was 1 with a range of 1–2. Seven patients (16.3%) of patients regained a performance status $\le$ 1. Factors associated with an improvement in performance status within the radiation therapy group were extent of resection (biopsy only vs. partial resection vs. gross total resection, *p* = 0.039), combined use of systemic therapy (*p* = 0.001) and delivered radiotherapy dose (< 40 vs ≥ 40 Gy, *p* = 0.007). It has to be noted that in 75% of patients with a radiotherapy dose < 40 Gy, radiotherapy had to be terminated early. The majority of patients with an improvement in performance status had a gross total resection, received systemic therapy and all had received a radiation dose of ≥ 40 Gy. Age, MGMT status and the initial performance status were not associated with improvement in performance status after radiotherapy.

## Discussion

This retrospective single-institution analysis addressed the value of radiotherapy compared to best supportive care in glioblastoma patients with poor postoperative performance status in the first-line setting.

Performance status is a well-documented prognostic factor for overall survival in glioblastoma patients, the most important factors being age (< 50 vs. ≥ 50 years) (Curran et al. 1993; Li et al. 2011) or type of surgery (Gross total resection/partial resection vs. biopsy) (Scott et al. 2012). However, with regard to the optimal treatment recommendation of patients in poor performance status, reliable data is sparse (Curran et al. 1993; Lutterbach et al. 2003; Li et al. 2011; Scott et al. 2012; Stark et al. 2012; McNamara et al. 2014; Gately et al. 2016; Glynn, Rangaswamy et al. 2019). The median overall survival of patients with poor performance status is short with 2.3–6.6 months from the time of diagnosis, although the definition of poor performance status is inconsistent. In a cohort published by Scott et al., patients with biopsy only and a KPS < 70% had a median OS of 2.3 months. Patients with more extensive surgeries had a better overall survival, independent of performance status (Scott et al. 2012). Marina et al. analyzed data of patients with a KPS < 50% in a cohort with about half of patients biopsied only, and 64% of patients receiving radiotherapy, and reported a median overall survival from the date of the most excessive surgery of 2.3 month, too (Marina et al. 2011).

In our cohort, the median survival from diagnosis was 27 weeks in all patients treated with radiotherapy, and 13 weeks in the biopsy only followed by radiotherapy group. Survival from the end of radiotherapy was median 13 weeks for all patients treated with radiotherapy and 8 weeks for biopsy followed by radiotherapy. This indicates patients treated with biopsy only are at increased risk of early death irrespective of post-biopsy radiotherapy. Admittedly, our radiotherapy group comprised more patients with gross total resection compared to the best supportive care group. Yet, without post-surgery radiotherapy, survival does not increase with gross total resection compared to biopsy only, underlining the role of radiotherapy (Fig. 2A, B), and the median overall survival from diagnosis was significantly better in the radiotherapy group than in the control group (10 weeks) irrespective of performance status or extent of surgical resection. Landmark analyses at 4 and 8 weeks confirmed the survival benefit in the radiotherapy group (Fig. 3).

In patients with poor performance status, survival is only one aspect to consider. Another important question is whether impaired patients may benefit from radiation therapy in terms of performance status improvement. Very few data are available on this topic, and especially prospective data is rare. Retrospective data and data from a phase II trial suggest that KPS might improve by 10–30% in up to 70% patients with radiotherapy, but overall patient numbers are small (Gallego Perez-Larraya et al. 2011; Marina et al. 2011; Reyngold et al. 2012). In this analysis, 30% of patients had an improvement in performance status of 1–2 after radiotherapy, consistent with previous data. One fifth of our patient population regained an ECOG ≥ 1, allowing for self-care, and therefore representing a factor of autonomy for the patient.

Another factor to consider is treatment-induced toxicity. Radiation-induced toxicity for glioblastoma patients is usually low, e.g., 8% ≥ G3 toxicity in the radiation arm (Malmstrom et al. 2012; Wick et al. 2012) in a group of elderly, therefore more vulnerable, patients. This is confirmed in our study with no case of grade 3 or higher radiation-induced toxicity. Treatment with TMZ, either as monotherapy or with radiation, causes mostly hematologic ≤ G3 toxicity in up to 25% of patients (Stupp et al. 2005; Gallego Perez-Larraya et al. 2011; Malmstrom et al. 2012).

Short course radiation treatment is the standard of care in elderly and poor-performance status patients with unmethylated MGMT-promotor (Guedes de Castro et al. 2017; Weller et al. 2017), which limits the total treatment time to 3 weeks and 15 radiotherapy sessions. All patients in our study were treated with hypo-fractionated radiotherapy; however, it was interesting that the benefit of radiotherapy was restricted to patients treated with 40.05 Gy in 15 fractions, whereas no benefit was observed for lower-dose radiotherapy. Best-supportive care or shorter fractionated radiotherapy should, therefore, be considered for patients not eligible for a full-course radiotherapy of 15 fractions over three weeks.

Despite absolute overall survival is short in glioblastoma patients with poor performance status, as reported above, radiotherapy was well-tolerated as delivered over maximum three weeks and appears favorable considering that median overall survival is prolonged by a factor of almost three. However, careful and balanced patient information about the absolute and relative benefits of surgery and postoperative radiotherapy and chemotherapy is mandatory.

Limitations of this study include the retrospective nature of this small population with heterogeneous treatment, including combination of chemotherapeutics with radiotherapy as part of the first-line treatment. However, there was no significant imbalance with regard to MGMT promotor methylation as predictive marker in the radiotherapy plus systemic treatment group, which could have indicated a better response to additional chemotherapy. Also, lack of patient-reported outcomes in this retrospective analysis precludes a statement on subjective benefits and quality of life in this specific patient population, therefore omitting a relevant patient-centered outcome surrogate. Still, the inclusion of relevant numbers of patients biopsied only, in both radiotherapy and control group (25 vs. 26 patients, respectively), provides more information on this patient group with poor predictors of outcome that may benefit with regard to the performance status improvement.

In conclusion, this retrospective single-institution analysis observed a significant and clinically relevant overall survival benefit of radiotherapy in glioblastoma patients with poor performance status. However, considering the short absolute overall survival, especially in patients not eligible for neurosurgical resection, the potential benefit of prolonged survival must be carefully weighted against the large portion of extended lifespan being spent under treatment. Better prognostic and predictive factors for patient selection are urgently needed. Most importantly, quality-of-life and patient-reported outcome need to be considered in addition to sole overall survival and should be incorporated not only into clinical studies, but also as routine practice, including patients in reduced performance status who are usually excluded from study populations (Coomans et al. 2019; Hertler et al. 2020).

## Supplementary Information

Below is the link to the electronic supplementary material.

Supplementary file1 Supplementary Figure Repartition of tumor location by hemisphere (A, B) or lobe (C, D) in the radiotherapy group (left panel) or the control group (right panel) by biopsy or resection. (PDF 242 kb)

## Declarations

### Data availability

No data are available.
