# Supplementary figures and images for: Radiotherapy for glioblastoma patients with poor performance status
Source: J Cancer Res Clin Oncol. 2021 Aug 26;148(8):2127–36. doi: 10.1007/s00432-021-03770-9 (PMC9293860; doi:10.1007/s00432-021-03770-9)

# Supplementary File

Radiotherapy group

Control group

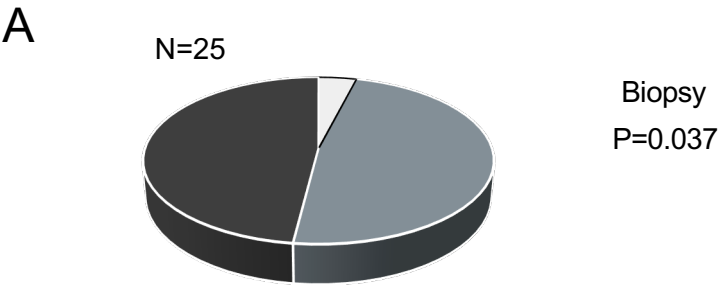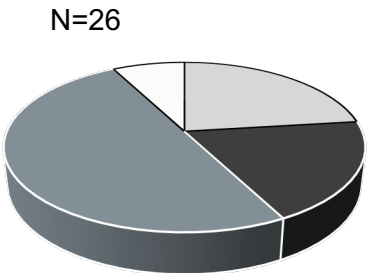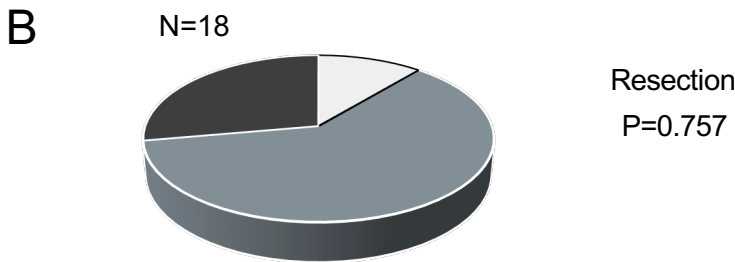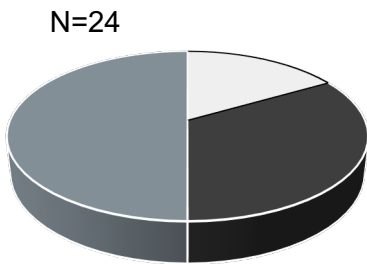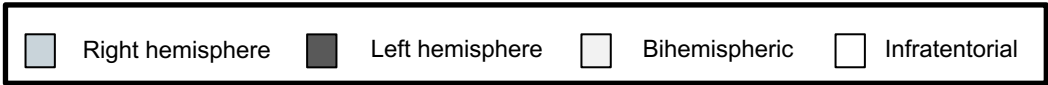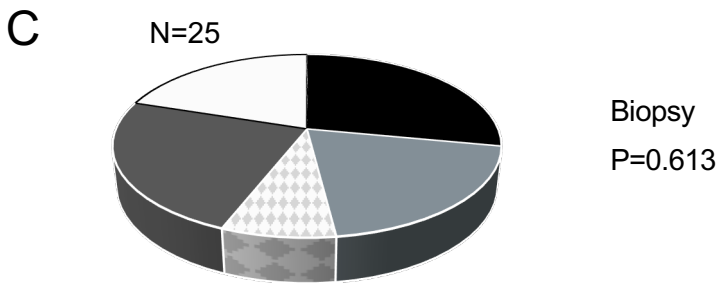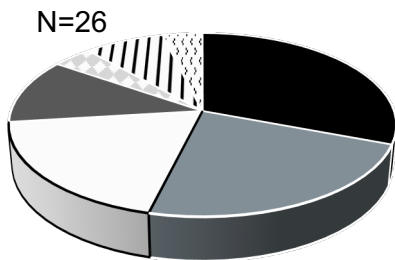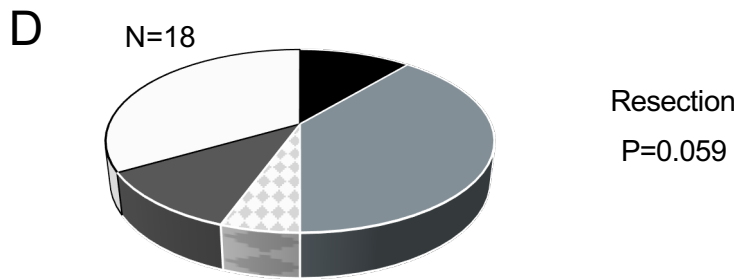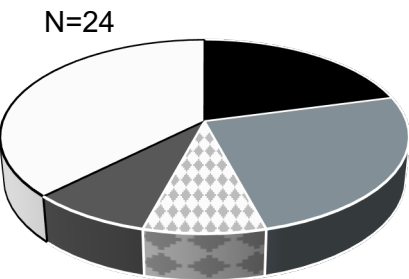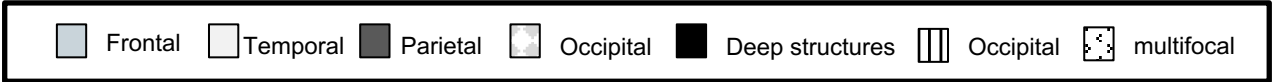

Supplement: Supplementary file 1 — Supplementary file1 Supplementary Figure Repartition of tumor location by hemisphere (A, B) or lobe (C, D) in the radiotherapy group (left panel) or the control group (right panel) by biopsy or resection. (PDF 242 kb) [file 432_2021_3770_MOESM1_ESM.pdf]
